# Supplementary material for: LINC00958 and HOXC13-AS as key candidate biomarkers in head and neck squamous cell carcinoma by integrated bioinformatics analysis
Source: PeerJ. 2020 Feb 13;8:e8557. doi: 10.7717/peerj.8557 (PMC7024572; doi:10.7717/peerj.8557)
Supplement: Table S3 [file peerj-08-8557-s003.docx]

**Supplement Table 2 *P* value of different origins in HNSCC and normal samples**

| Tissue | LINC00958 | HOXC13-AS |
| --- | --- | --- |
| Normal | 0.51 | 0.06889 |
| HNSCC | 0.2959 | 0.07667 |
