# Supplementary figures and images for: LINC00958 and HOXC13-AS as key candidate biomarkers in head and neck squamous cell carcinoma by integrated bioinformatics analysis
Source: PeerJ. 2020 Feb 13;8:e8557. doi: 10.7717/peerj.8557 (PMC7024572; doi:10.7717/peerj.8557)

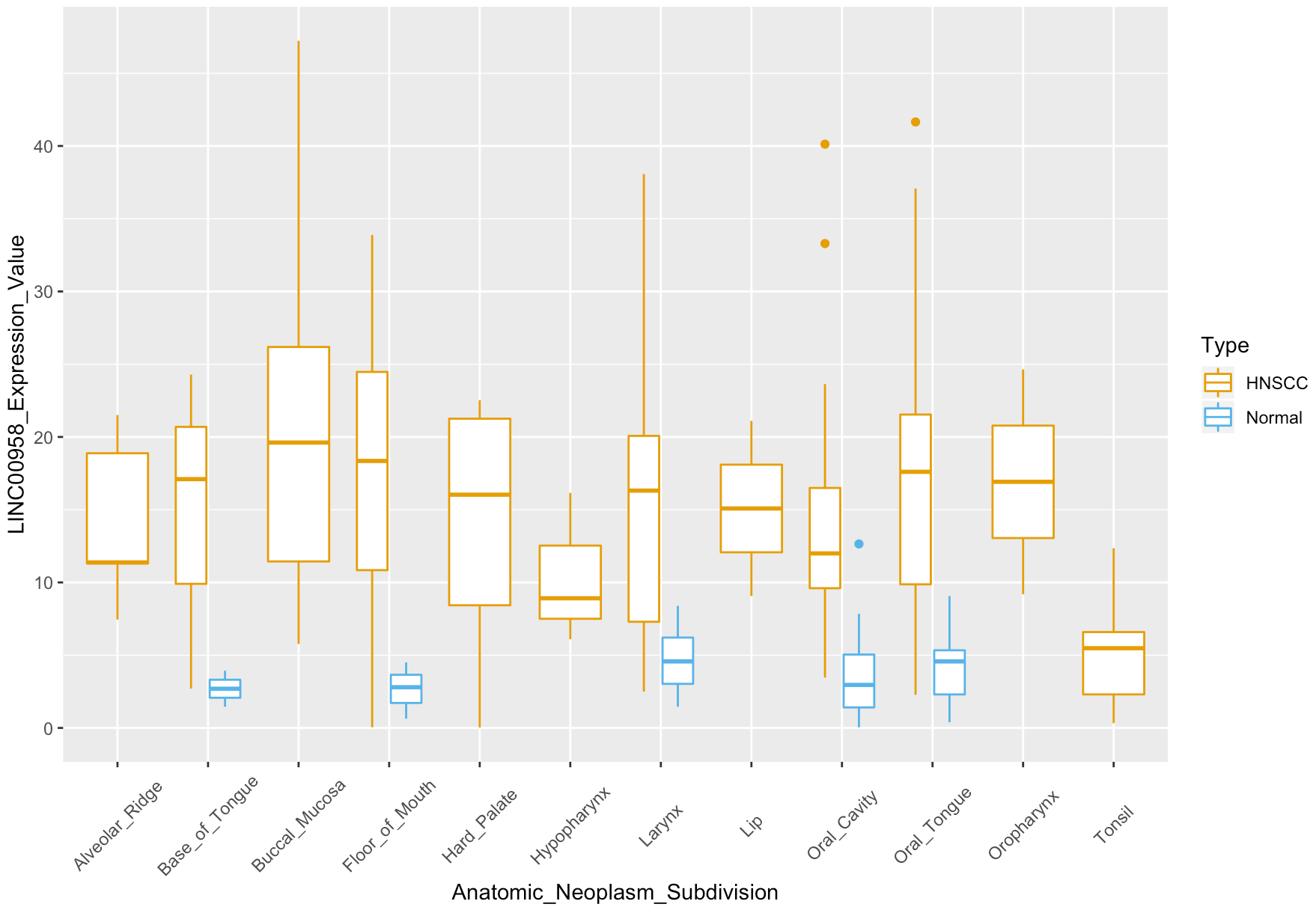

Supplement: Figure S1 [file peerj-08-8557-s004.png]

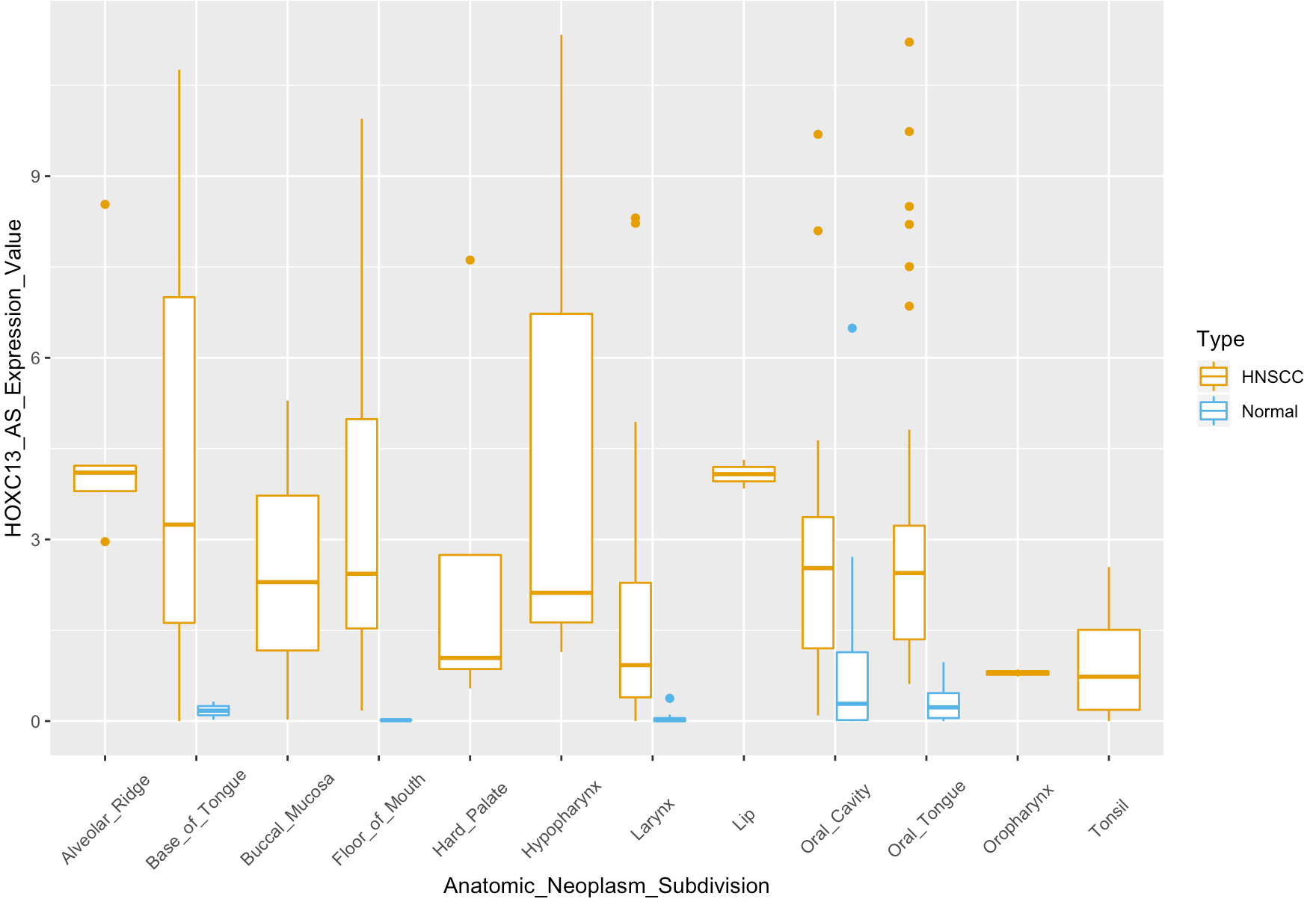

Supplement: Supplemental Information 5 [file peerj-08-8557-s005.png]
